# ﻿ *Alternariaphoenicis* sp. nov. and *Alternariaouedrighensis* sp. nov. (Pleosporales, Pleosporaceae): Two new species associated with leaf spot and blight diseases of date palm (*Phoenixdactylifera* L.)

**DOI:** 10.3897/mycokeys.120.144245

**Published:** 2025-08-15

**Authors:** Youssef Djellid, Alla Eddine Mahamedi, Milan Spetik, Eliska Hakalová, Ales Eichmeier, Micael Ferreira Mota Gonçalves, Fouad Lamghari, Maryam Ali Saeed Mohamed Al Hmoudi, Akila Berraf-Tebbal

**Affiliations:** 1 Laboratoire de Biologie des Systèmes Microbiens (LBSM), Ecole Normale Supérieure Cheikh Mohamed El Bachir El Ibrahimi de Kouba, 16308 Vieux-Kouba, Alger, Algeria Laboratoire de Biologie des Systèmes Microbiens (LBSM), Ecole Normale Supérieure Cheikh Mohamed El Bachir El Ibrahimi de Kouba Alger Algeria; 2 Département de Biologie, Faculté des Sciences de la Nature et de la Vie, et des Sciences de la Terre, Université de Ghardaia, 47000 Ghardaïa, Algeria Université de Ghardaia Ghardaïa Algeria; 3 Mendeleum - Institute of Genetics, Faculty of Horticulture, Mendel University in Brno, Valticka 334, 69144, Lednice, Czech Republic Mendel University in Brno Lednice Czech Republic; 4 CESAM, Departamento de Biologia, Universidade de Aveiro, 3810-193 Aveiro, Portugal Universidade de Aveiro Aveiro Portugal; 5 Fujairah Research Centre, Sakamkam Road, Fujairah 00000, United Arab Emirates Fujairah Research Centre Fujairah United Arab Emirates

**Keywords:** *Alternaria*, leaf spot and blight diseases, *Phoenixdactylifera* L., phylogeny, taxonomy

## Abstract

Date palm (*Phoenixdactylifera* L.) is one of the oldest fruit crops grown in the semi-arid and arid regions, playing significant ecological, environmental and socio-economic roles. Recently, palm leaf spot and blight diseases have indeed emerged as significant threats to phoeniciculture. They reduce yield and quality of dates leading to economic losses. Therefore, a survey was conducted in four palm groves located in the Biskra and Ghardaia provinces of Algeria. This investigation revealed two new *Alternaria* species associated with leaf spot and blight symptoms on date palm. These newly identified species are designated as *A.phoenicis***sp. nov.** and *A.ouedrighensis***sp. nov.**, which belong to the *Ulocladioides* and *Embellisia* sections, respectively. The isolates were phylogenetically identified using the key genetic markers of the genus including the large subunit ribosomal DNA (LSU), internal transcribed spacer region of the ribosomal RNA (ITS), glyceraldehyde-3-phosphate dehydrogenase (*GAPDH*), RNA polymerase II subunit (*RPB2*), translation elongation factor (*TEF1*) and plasma membrane (*ATPase*) genes and illustrated based on the morphological characteristics.

## ﻿Introduction

The date palm (*Phoenixdactylifera* L.) is a dioecious perennial monocot in the Arecaceae family, which comprises around 200 genera and 1500 species (Dawson 1982). It is a vital crop in desert regions, serving as a primary source of food and trade from North Africa to India and across other subtropical areas (Erskine et al. 2004). Notably, Algeria stands as the world’s third-largest date producer, generating over 1.3 million tonnes annually, where date palms underpin both traditional and modern Saharan agriculture (FAO 2023). However, despite its economic significance, date palms are vulnerable to various pathogenic fungi that can severely damage their stem, leaves, fruit, and root, leading to substantial yield reductions (Bokhary 2010; El-Juhany 2010).

Among the fungi that impact date palms, *Alternaria* emerges as a particularly associated group with leaf spots and blight diseases in the Middle East regions (El-Juhany 2010; Al-Sadi et al. 2012; Al-Nadabi et al. 2018). *Alternaria*, a genus in the family Pleosporaceae, order Pleosporales, and phylum Ascomycota, was first described by Nees in 1816 with *Alternariatenuis* designated as the type species. Since then, the taxonomy of *Alternaria* has undertaken significant revisions leading to the identification of numerous new species. Presently, the genus comprises more than 360 species encompassing 29 sections (Simmons 2007; Woudenberg et al. 2013; Wijayawardene et al. 2020; Li et al. 2023).

Species of *Alternaria* occupy a wide range of ecological niches, occurring as endophytes within apparently asymptomatic plant tissues, saprobes on various substrates such as dead vegetation, paper, and food, and as pathogens that impact both plants and animals, including humans, worldwide. This adaptability enables them to thrive in diverse environments and interact with a wide range of hosts (Blodgett et al. 2000; Larran et al. 2001; Feng et al. 2011; Qi et al. 2012; Li et al. 2023; He et al. 2024).

The *Alternaria* genus consists of several phytopathogenic species that cause diseases in a wide array of plants around the world, affecting key crops such as cabbage, cauliflower, tomato, carrot, wheat, cucurbits and date palm (Chaerani and Voorrips 2006; Logrieco et al. 2009; Rahimloo and Ghosta 2015; Al-Nadabi et al. 2018; Jayawardena et al. 2019). These pathogens primarily induce leaf spots and defoliation, characterized by necrotic lesions and yellowing on leaves (Mac Kinon et al. 1999). They can also infect various plant parts, including seedlings and fruits, leading to significant pre- and post-harvest losses (Thomma 2003; Lawrence et al. 2016). Furthermore, *Alternaria* species are recognized as seed-borne pathogens and are known for producing harmful secondary metabolites, including phytotoxins and mycotoxins (Thomma 2003; Simmons 2007; Gilardi et al. 2015; Lawrence et al. 2016; Chalkley 2020).

*Alternaria* genus includes morphologically diverse species traditionally identified by reproductive structures, sporulation patterns, and host interactions. However, taxonomic classification has been debated due to species complexes and morphological variability influenced by environmental conditions and host specificity (Elliot 1917; Fries 1832; Neergaard 1945; Joly 1964; Simmons 1967). Afterward, Simmons introduced practical criteria to standardize taxonomic concepts for *Alternaria* species, focusing on colony and conidial morphology (Simmons 2007). Therefore, in recent years, DNA sequencing of conserved loci has massively improved the knowledge of fungal phylogeny. Several studies have shown that phylogenetic analysis becomes a reliable approach for species-level identification. The multilocus phylogeny using genetic regions such as ITS, LSU, *TEF1*, *RPB2*, *GAPDH* and *Alt-a1* combined with morphological data are frequently used to resolve the taxonomy and identification of *Alternaria* taxa. Thus, new species are increasingly described (Woudenberg et al. 2013; Al Ghafri et al. 2019; Li et al. 2023; Aung et al. 2024; He et al. 2024; Jayawardena et al. 2025).

During an investigation of *Alternaria* species in Algeria, two new taxa were isolated from date palm (*Phoenixdactylifera* L.). This study used a polyphasic approach, integrating both morphological and phylogenetic analyses, to characterize these newly introduced taxa.

## ﻿Materials and methods

### ﻿Isolation and morphological studies

**Figure 1.**
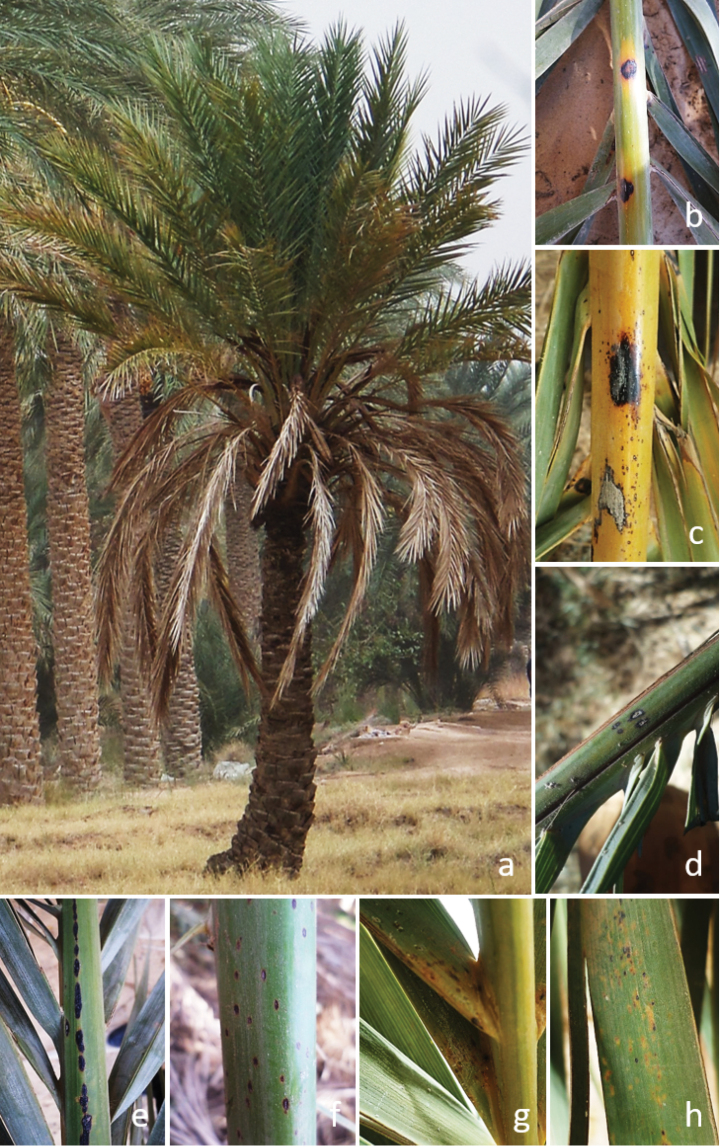
Date palm tree with decline symptoms (a), rachis (b–f) and leaflets (g, h) spots.

During 2017, a set of 40 samples comprising leaves, rachises, and leaflets with spot lesions was collected from date palm trees in Ghardaia and Bechar provinces, Algeria (Fig. 1). Plant material was carefully enclosed in paper bags and transported to the laboratory. Subsequently, isolations were made from the margin of symptomatic tissues. Small pieces (approx. 5 mm^2^) of rachis and leaflets were surface sterilized in 5% sodium hypochlorite (NaOCl) for 8 and 4 min, respectively. They were rinsed thrice with sterile distilled water, then dried with sterilized filter paper and placed onto the surface of potato dextrose agar (PDA, Difco Laboratories). Plates were incubated at 25 °C until fungal growth was perceived. The mycelium emerged from the fragments of the tissues were transferred to new PDA plates and incubated under the same conditions.

Colony growth characteristics including surface and reverse appearance of the culture were recorded after 7 days of incubation on 90 mm diameter PDA Petri plates at 25 °C in darkness, following Li et al. (2022) and Luo et al. (2022). Growth characteristics were determined on PDA plates incubated at different temperatures from 5–40 °C at 5 °C intervals in the dark. Reference strains and specimens are maintained at the Fungal Biodiversity Centre (CBS) and MEND-F fungal collections.

Fungal colonies were subcultured onto water agar medium, supplemented with autoclaved poplar twigs to enhance sporulation (Santos and Phillips 2009). The cultures were maintained on a laboratory bench at approximately 20–25 °C, where they were exposed to diffused daylight. After two weeks, observations of micromorphological features including conidial size, shape, colour, striation, septation, conidiophores and conidiogenous cells mounted into 100% lactic acid, were made using a Nikon Eclipse 80i microscope. Photographs and measurements of fungal structures mounted in 100% lactic acid were taken with a Nikon DSRi1 camera and the software NIS-Elements D (Nikon). Thirty measurements per structure were performed and presented in the quantitative format “(min–) low – up (–max) × (min–) low – up (–max) µm (av. Length mean ± SD × Width mean ± SD µm)”, with full observed ranges (minimum–maximum), typical ranges (low–up), and mean ± standard deviation.

### ﻿DNA extraction and sequencing

Genomic DNA of our isolates was extracted from 7-day-old mycelium grown on PDA at 25 °C. The NucleoSpin Tissue kit (Macherey-Nagel, Düren, Germany) was used according to the manufacturer’s instructions (https://www.mn-net.com).

Polymerase chain reaction amplifications of the large subunit ribosomal DNA (LSU), internal transcribed spacer of ribosomal DNA (ITS), parts of glyceraldehyde-3-phosphate dehydrogenase (*GAPDH*), RNA polymerase II subunit (*RPB2*), translation elongation factor (*TEF1*) and plasma membrane adenosine triphosphatase (*ATPase*) genes were performed using primer pairs (Table 1). Polymerase chain reaction (PCR) mixtures and amplification conditions were conducted following the protocols described by Berbee et al. (1999) and Woudenberg et al. (2013). PCR mixture contained 10 μM of primer, 200 μMdNTP, 1×Taq reaction buffer, 2 Units of AmpliTaq-DNA polymerase, 2.5 mMMgCl_2_ and 10 ng of template DNA for a final reaction volume of 25 μl. After amplification, the obtained PCR amplicons were purified and sequenced by the company Eurofins (Germany).

**Table 1.** Primers used for PCR amplification and sequencing of *Alternaria* genes.

Genes	Primers	References
ITS	ITS1	White et al. 1990
	ITS4	
*TEF1*	EF1-728F	Carbone and Kohn 1999
	EF1-986R	
*RPB2*	RPB2–5F2	Sung et al. 2007
	RPB2–7cR	Liu et al. 1999
*GAPDH*	gpd 1	Berbee et al.1999
	gpd 2	
*ATPase*	ATPDF1	Lawrence et al. 2013
	ATPDR1	
LSU	LROR	Rehner and Samuels 1994
	LR7	Vilgalys and Hester 1990

### ﻿Phylogenetic analysis

The obtained sequences of ITS, LSU, *GAPDH*, *RPB2*, *TEF1* and *ATPase* regions were checked and manually adjusted, when necessary, using BioEdit Sequence Alignment Editor v.7.0.4.1 (Hall 1999). Sequence alignments were conducted through the online version of the multiple sequence alignment program (MAFFT) v.7 (Katoh et al. 2019) using the default settings. Newly generated sequences were deposited in GenBank (Table 2).

**Table 2.** *Alternaria* species used for phylogenetic analysis. Newly generated sequences are indicated in bold face.

Species	Strain No	Section	Host	Country	GenBank accession numbers					
					*GAPDH*	*RPB2*	*TEF1*	ITS	LSU	*ATPase*
*A.abundans*	CBS 534.83	*Chalastospora*	*Fragaria* sp.	New Zealand	KC584154	KC584448	KC584707	JN383485	KC584323	JQ671802
*A.allii-tuberosi*	CBS 124112	*Ulocladioides*	-	China	KF533907	-	-	-	-	-
*A.alstroemeriae*	MAFF 241374	*Alternaria*	*Alstroemeria* sp.	Japan	AB744034	LC275231	LC275050	AB678214	-	-
*A.alternantherae*	CBS 124392	*Alternantherae*	*Solanummelongena*	China	KC584096	KC584374	KC584633	KC584179		-
*A.alternariae*	CBS126989	*Ulocladium*	*Daucuscarota*	USA	AY376329	KC584470	KC584730	AY376642	KC584346	-
*A.alternata*	CBS 102598	*Alternaria*	-	-	KP124184	KP124797	KP125105	MH862798	MH874394	JQ671884
	CBS 916.96	*Alternaria*	*Arachishypogaea*	India	AY278808	KC584375	KC584634	AF347031	-	-
	CBS 918.96	*Alternaria*	*Dianthus* sp.	UK	AY278809	KC584435	KC584693	AF347032	KC584311	-
*A.anthropophila*	FMR 16235	*Infectoriae*	Human	Spain	LR537034	LR537040	LR537046	LR537444	-	LR537052
*A.arborescens*	CBS 102605	*Alternaria*	*Lycopersicon* sp.	USA	AY278810	KC584377	KC584636	AF347033	NG_069124	-
*A.argyranthemi*	CBS 116530	-	*Argyranthemum* sp.	New Zealand	KC584098	KC584378	KC584637	KC584181	KC584254	-
*A.armoraciae*	CBS 118702	*Chalastospora*	*Armoraciarusticana*	New Zealand	KC584099	KC584379	KC584638	KC584182	KC584255	LR134098
*A.aspera*	CBS 115269	*Pseudoulocladium*	*Pistaciavera*	Japan	KC584166	KC584474	KC584734	KC584242	KC584349	-
*A.atra*	CBS 195.67	*Ulocladioides*	Soil	USA	KC584167	KC584475	KC584735	AF229486	KC584350	JQ671833
	CBS 102060	*Ulocladioides*	Soil	Canada	KC584174	KC584484	KC584744	FJ266486	MH874371	JQ671837
*A.atrobrunnea*	FMR 16868	*Infectoriae*	Human skin lesion	Spain	LR537039	LR537044	LR537051	LR537033	-	LR537057
*A.betae-kenyensis*	CBS 118810	*Alternaria*	*Betavulgaris*	Kenya	KP124270	KP124888	KP125197	KP124419	NG_069256	MH10180
*A.bornmuelleri*	DAOM 231361	*Undifilum*	*Securigeravaria*	Austria	FJ357305	KC584491	KC584751	FJ357317	KC584366	JQ671791
*A.botryospora*	CBS 478.90	*Embellisioides*	*Leptinelladioica*	New Zealand	AY278831	KC584461	KC584720	AY278844	KC584336	JQ671779
*A.brassicicola*	CBS 118699	*Brassicicola*	*Brassicaoleracea*	USA	KC584103	KC584383	KC584642	JX499031	KC584259	-
*A.brassicifolii*	CNU 111118	-	*Brassicapekinensis*	South Korea	KM821537	-	-	JQ317188	-	KY412558
*A.breviconidio-phora*	MFLUCC 21-0786	*Alternaria*	*Digitalis* sp.	Italy	OK236604	OK236651	OK236698	MZ621997	MZ621944	-
*A.breviramosa*	CBS 121331	*Chalastospora*	*Triticum* sp.	Australia	KC584148	KC584442	KC584700	FJ839608	KC584318	LR134099
*A.brevirostra*	MFLUCC 21-0129	*Radicina*	*Plantago* sp.	Italy	OK236619	OK236666	OK236717	MZ622015	-	-
*A.burnsii*	CBS 107.38	*Alternaria*	*Cuminumcyminum*	India	JQ646305	KP124889	KP125198	KP124420	NG_069257	JQ671860
*A.cantlous*	CBS 123007	*Ulocladioides*	*Cucumismelo*	China	KC584171	KC584479	KC584739	KC584245	MH874786	-
*A.alternarina*	CBS 119396	*Infectoriae*	*Avenasativa*	USA	JQ646289	JQ905199	LR134367	JQ693648		JQ671817
*A.celosiicola*	MAFF 243058	*Alternantherae*	*Celosiaargentea*	Japan	AB744033	LC476781	LC480205	AB678217	-	-
*A.oblongo-obovoidea*	CBS 126317	*Ulocladioides*	-	China	FJ266494	-	-	-	-	-
	CBS 201.67	*Ulocladioides*	-	China	FJ266495	JQ905212	JQ672439	-	-	JQ671839
*A.caespitosa*	CBS 177.80	*Infectoriae*	-	Spain	KC584178	KC584492	KC584752	MH861255	KC584367	-
*A.cantlous*	MF-P 262011	*Ulocladioides*	Carrot seed	Russia	MW658286	OQ262917	OQ262897	-	-	-
*A.capsici-annui*	CBS 504.74	*Ulocladium*	*Capsicumannuum*	-	KC584105	KC584385	KC584644	KC584187	KC584261	KC584385
*A.caricis*	CBS 480.90	*Nimbya*	*Carexhoodia*	USA	AY278826	KC584467	KC584726	AY278839	KC584342	JQ671780
*A.castaneae*	CBS 124390	*Ulocladioides*	-	-	KF533902	-	-	-	-	-
*A.cetera*	EGS 41.072	*Chalastospora*	*Elymusscabrus*	Australia	AY562398	KC584441	KC584699	JN383482	KC584317	JQ671801
*A.chartarum*	MAFF 246888	*Pseudoulocladium*	*Capsicumannuum*	Japan	LC482041	LC476826	LC480245	LC440618	KC584356	-
	CBS 115269	*Pseudoulocladium*	*Pistaciavera*	Japan	KC584166	KC584474	KC584734	NR_145169	NG_069147	
*A.cheiranthi*	CBS 109384	*Cheiranthus*	*Cheiranthuscheiri*	Italy	KC584107	KC584387	KC584646	AF229457	KC584263	-
*A.chlamydospora*	CBS 491.72	*Phragmosporae*	Soil	Egypt	KC584108	KC584388	KC584647	KC584189	KC584264	JQ671786
*A.chlamydosporigena*	CBS 341.71	*Embellisia*	Air	USA	KC584156	KC584451	KC584710	KC584231	KC584326	-
*A.conjuncta*	CBS 196.86	*Infectoriae*	*Pastinacasativa*	Switzerland	AY562401	KC584390	KC584649	FJ266475	KC584266	JQ671824
*A.consortialis*	CBS 104.31	*Ulocladioides*	*Cucumber leaf*	Russia	KC584173	KC584482	KC584742	MH855147	MH866597	-
	CBS 121493	*Ulocladioides*	*Brassicarapasubsp.Pekinensis*	China	KC584170	KC584478	KC584738	NG_067641	KC584353	-
	CBS 101229	*Ulocladioides*	*Cucumissativus*	New Zealand	FJ266498	KC584485	KC584745	KC584618	KC584360	JQ671838
	CBS 483.81	*Ulocladioides*	*Cucumissativus*	New Zealand	AY562418	KC584483	KC584743	KC584616	KC584358	-
	CBS 202.67	*Ulocladioides*	-	USA	KC584177	KC584490	KC584750	NR_103600	NG_069728	JQ671835
	CBS 198.67	*Ulocladioides*	Soil	USA	KC584169	KC584477	KC584737	KC584610	KC584352	-
*A.cumini*	CBS 121329	*Eureka*	*Cuminumcyminum*	India	KC584110	KC584391	KC584650	KC584191	KC584267	-
*A.cylindrica*	MAFF 246770	*Alternaria*	*Petuniaatkinsiana*	USA	LC482006	LC476791	LC480211	LC440584	-	-
*A.dactylidicola*	MFLUCC 15-0466	*Infectoriae*	*Loliummultiflorum*	China	MK051155	MK051157	-	NG_063635	NG_069434	-
*A.daucifolii*	CBS 118812	*Alternaria*	*Daucuscarota*	USA	KC584112	KC584393	KC584652	KC584193	NG_069131	MH101801
*A.dennisii*	CBS 476.90	-	*Seneciojacobaea*	Isle of Man	JN383469	KC584454	KC584713	JN383488	KC584329	-
*A.dianthicola*	CBS 116491	*Dianthicola*	*Dianthus* sp.	New Zealand	KC584113	KC584394	KC584653	KC584194	KC584270	-
*A.dongshanqi-aoensis*	DSQ2.2	*Alternaria*	Chinese fir leaf	China	OR252415	OR252511	OR233901	OR229433	OR229638	-
*A.eichhorniae*	CBS 489.92	*Alternaria*	*Eichhornia*sp.	India	KP124276	KP124895	KP125204	KC146356	KP124579	MH101806
*A.elegans*	CBS 109159	*Dianthicola*	*Lycopersiconesculentum*	Burkina Faso	KC584114	C584395	KC584654	KC584195	KC584271	-
*A.embellisia*	CBS 339.71	*Embellisia*	*Alliumsativum*	USA	KC584155	KC584449	KC584708	KC584230	KC584324	-
*A.ershadii*	IRAN 3275C	*Pseudoalternaria*	Wheat	Iran	MK829645	-	-	MK829647	-	MK829643
*A.ethzedia*	CBS 197.86	*Infectoriae*	*Brassicanapus*	Switzerland	AY278795	KC584398	KC584657	NG_062882	NG_069134	JQ671805
*A.eupatoriicola*	MFLUCC 21-0122	*Alternaria*	*Eupatorium* sp.	Italy	OK236589	OK236636	OK236683	MZ621982	MZ621929	-
*A.euphorbiacola*	CBS 119410	*Radicina*	*Euphorbia* sp.	USA	KJ718018	-	KJ718521	KJ718173	-	KJ718346
*A.eureka*	CBS 193.86	*Eureka*	*Medicagorugosa*	Australia	JN383471	KC584456	KC584715	JN383490	KC584331	JQ671771
*A.gaisen*	CBS 632.93	*Alternaria*	*Pyruspyrifolia*	UK	KC584116	KC584399	KC584658	KC584197	KC584275	-
*A.geniostomatis*	CBS 118701	*Eureka*	*Geniostoma* sp.	N. Zealand	KC584117	KC584400	KC584659	KC584198	KC584276	-
*A.geophila*	CBS 101.13	*Alternaria*	Peat soil	Switzrland	KP124244	KP124862	KP125170	KP124392	-	KP124862
*A.gomphrenae*	MAFF 246769	*Alternantherae*	*Gomphrenaglobosa*	Japan	LC481999	LC476782	LC480206	LC440579	-	-
*A.graminicola*	CBS 119400	*Infectoriae*	Solanaceae	Algeria	MK904514	LR134180	LR134249	NR_136024		MK913529
*A.guarroi*	FMR 16556	*Infectoriae*	Human skin lesion	Spain	LR537037	LR537045	LR537050	LR537031	-	LR537056
*A.halotolerans*	CBS 146348	*Infectoriae*		Qatar	KY387604	-	KY387608	KY387606	-	-
*A.helianthiinfi-ciens*	CBS 117370	*Helianthiinficientes*	*Helianthusannuus*	UK	KC584119	KC584402	KC584661	KC584200	KC584279	-
*A.hyacinthi*	CBS 416.71	*Embellisioides*	*Hyacinthus*sp.	Netherlands	KC584158	KC584457	KC584716	KC584233	KC584332	JQ671778
*A.indefessa*	CBS 536.83	*Cheiranthus*	Soil	USA	KC584159	KC584458	KC584717	KC584234	KC584333	JQ671831
*A.infectoria*	CBS 210.86	*Infectoriae*	*Triticumaestivum*	UK	DQ323697	KC584404	KC584662	DQ323697	KC584280	-
*A.hordeiaus-tralica*	CBS 119402	*Infectoriae*	*Hordeumvulgare*	Australia	JQ646283	LR134179	LR134243	NR_136018	-	JQ671811
*A.inflata*	FMR 16477	*Pseudoalternaria*	-	-	MT108483	-	-	MT109376		MT108479
*A.intercepta*	CBS 119406	*Infectoriae*	*Viburnum* sp.	Netherlands	FJ214831	LR134170	FJ214927	NR_135957		JQ671826
*A.juxtiseptata*	CBS 119673	*Gypsophilae*	*Gypsophila* sp.	Australia	KC584122	KC584406	KC584664	KC584202	KC584282	-
*A.lathyri*	MFLUCC 21-0140	*Alternaria*	*Lathyrus* sp.	Italy	OK236581	OK236628	OK236675	MZ621974	MZ621921	-
*A.leucanthemi*	CBS 421.65	*Teretispora*	*Chrysanthemummaximum*	Netherlands	KC584164	KC584472	KC584732	KC584240	KC584347	-
*A.limaciformis*	CBS 481.81	*Phragmosporae*	Soil	UK	KC584123	KC584407	KC584665	KC584203	KC584283	JQ671798
*A.limoniasperae*	CBS 102595	*Alternaria*	*Citrusjambhiri*	USA	AY562411	KC584408	KC584666	FJ266476	KC584284	JQ671879
*A.lolii*	CBS 115266	*Embellisioides*	*Loliumperenne*	N. Zealand	JN383473	KC584460	KC584719	JN383492	KC584335	JQ671774
*A.longipes*	CBS 540.94	*Alternaria*	*Nicotianatabacum*	USA	AY278811	KC584409	KC584667	AY278835	KC584285	-
*A.malicola*	CGMCC3.18704	*Ulocladioides*	-	China	MF426953	MF426957	MF426959	-	-	-
*A.malorum*	CBS 135.31	*Chalastospora*	-	-	JQ646278	JQ646481	JQ672413	JQ693638	-	JQ693638
*A.merytae*	CBS 119403	*Infectoriae*	-	USA	JQ646292	LR134119	LR134198	NR_136025		JQ671820
*A.metachroma-tica*	EGS 38.132	*Infectoriae*	-	China	AY762956	JQ905189	JQ672437	JQ693660	-	JQ671809
*A.microspora*	CBS 124391	*Ulocladioides*	-	-	KF533901	JQ905206	-	-	-	-
*A.momordicae*	YZU 161378	*Alternaria*	*Momordicacharantia*	China	OR887691	OR887689	OR887687	OR883774	-	-
*A.mouchaccae*	CBS 119671	*Phragmosporae*	Soil	Egypt	AY562399	KC584413	KC584671	KC584206	LC776460	JQ671799
*A.myanmarensis*	YZU 231736	*Alternaria*	*Helianthusannuus*	Myanmar	OR963612	PP508256	OR963615	OR897031	-	-
*A.nepalensis*	CBS 118700	*Japonicae*	*Brassica* sp.	Nepal	KC584126	KC584414	KC584672	KC584207	KC584290	-
*A.obclavata*	CBS 124120	*Chalastospora*	Air	USA	KC584149	KC584443	KC584701	KC584225	FJ839651	LR134100
*A.oblongoellip-soidea*	MFLUCC 22-0074	*Alternaria*	*Cichorium* sp.	Italy	OK236574	OK236621	OK236668	MZ621967	MZ621914	-
*A.obovoidea*	CBS 101229	*Ulocladioides*	*Cucumissativus*	New Zealand	FJ266498	KC584485	KC584745	FJ266487	KC584360	-
*A.omanensis*	SQUCC 15560	*Omanenses*	Dead wood	Oman	MK880900	MK880894	MK880897	MK878563	MK878557	-
	SQUCC 13580	*Omanenses*	Dead wood	Oman	MK880899	MK880893	MK880896	NG_074901	MK878556	-
***A.ouedrighensis***	**G92 = CBS 152587 = MEND-F-1168**	***Embellisia***	***Phoenixdactylifera* L.**	**Algeria**	**OP985422**	**OP985434**	**OP985443**	**OP295213**	**PQ349940**	-
*A.panax*	CBS 482.81	*Panax*	*Araliaracemosa*	USA	KC584128	KC584417	KC584675	KC584209	KC584293	-
*A.papavericola*	CBS 116608	*Crivellia*	*Papaverrhoeas*	Austria	FJ357299	KC584440	KC584698	FJ357311	KC584321	-
*A.paragomph-renae*	MAFF 246768	*Alternantherae*	*Gomphrena* sp.	Japan	LC482000	LC476783	LC480207	–	-	-
*A.parvicaespi-tosa*	LEP 014858	*Pseudoalternaria*	Diseased wheat heads	Iran	MF033842	-	-	MF033859	-	KJ908217
*A.penicillata*	CBS 116608	*Crivellia*	*Papaversomniferum*	USA	FJ357299	KC584440	KC584698	FJ357311	KC584316	-
*A.perpunctulata*	CBS 115267	*Alternantherae*	*Alternantheraphiloxeroides*	USA	KC584129	KC584418	KC584676	KC584210	KC584294	JQ671893
***A.phoenicis***	**G11 = CBS 152585 = MEND-F-1166**	***Ulocladioides***	***Phoenixdactylifera* L.**	**Algeria**	**OP985418**	**OP985431**	**OP985440**	**OP295203**	**PQ349938**	**OP985453**
	**A26**	***Ulocladioides***	***Phoenixdactylifera* L.**	**Algeria**	**OP985416**	**OP985432**	**OP985441**	**OP295200**	**PQ349937**	**OP985444**
	**A28**	***Ulocladioides***	***Phoenixdactylifera* L.**	**Algeria**	**OP985417**	**OP985433**	**OP985442**	**OP295201**	**PQ349939**	**OP985445**
*A.photistica*	CBS 212.86	*Panax*	*Digitalispurpurea*	UK	KC584131	KC584420	KC584678	KC584212	KC584296	JQ671807
*A.phragmos-pora*	CBS 274.70	*Phragmosporae*	Soil	Netherlands	JN383474	KC584462	KC584721	JN383493	KC584337	JQ671797
*A.preussii*	CBS 102062	*Ulocladioides*	-	USA	FJ266495	JQ905212	-	-	-	-
*A.phytolaccae*	MFLUCC 21-0135	*Radicina*	*Phytolaccaamericana*	Italy	OK236616	OK236663	OK236719	MZ622013	MZ621961	-
*A.qatarensis*	CBS 146387	*Chalastospora*	Sea water	Qatar	KY387603	-	KY387607	KY387605	KY781811	-
*A.radicicola*	NB830	*Embellisia*	*Daucuscarota*	Algeria	OP297090	OP320887	OP320893	OR085521	-	-
*A.radicina*	CBS 245.67	*Radicina*	*Daucuscarota*	USA	KC584133	KC584423	KC584681	NG067633	NG_069139	JQ671851
*A.rostroconidia*	MFLUCC 21-0136	*Alternaria*	*Arabis* sp.	Italy	OK236576	OK236623	OK236670	MZ621969	MZ621916	-
*A.salicicola*	MFLUCC 22.0072	*Alternaria*	*Aster* sp.	Russia	OK236606	OK236653	OK236700	MZ621999	MZ621946	-
*A.scirpicola*	CBS 481.90	*Nimbya*	*Scirpus* sp.	UK	KC584163	KC584469	KC584728	KC584237	KC584344	-
*A.selini*	CBS 109382	*Radicina*	*Petroselinum* sp.	Saudi Arabia	AY278800	KC584426	KC584684	AF229455	NG_069140	JQ671853
*A.slovaca*	CBS 567.66	*Infectoriae*	*Homosapiens*	Slovakia	KC584150	KC584444	KC584702	KC584226	KC584319	LR134368
*A.smyrnii*	CBS 109380	*Radicina*	*Smyrnium* sp.	UK	KC584138	KC584429	KC584687	AF229456	KC584305	-
*A.soliaridae*	CBS 118387	-	Soil	USA	KC584140	KC584431	KC584689	KC584218	KC584307	-
*A.subcucurbitae*	CBS 123376	*Ulocladioides*	-	China	KC584176	KC584488	KC584748	MH863292	MH874816	-
	CBS 121491	*Ulocladioides*	*Oxybasisglauca*	China	EU855803	KC584489	KC584749	NR_136053	NG_069148	-
*A.tellustris*	CBS 538.83	*Embellisia*	Soil	USA	AY562419	KC584465	KC584724	FJ357316	KC584340	JQ671794
*A.thalictrigena*	CBS 121712	-	*Thalictrum* sp.	Germany	KC584144	KC584436	KC584694	EU040211	KC584312	-
*A.tomato*	CBS 114.35	*Alternaria*	*Solanum* sp.	Unknown	KP124295	KP124916	KP125225	KP124446	KP124600	-
*A.torilis*	MFLUCC 14-0433	*Alternaria*	*Torilis* sp.	Italy	OK236593	OK236640	OK236687	MZ621986	MZ621933	-
*A.triangularis*	MAFF 246776	-	*Bupleurumrotundifolium*	Japan	LC482050	LC476837	LC480255	LC440629	-	-
*A.triticimacul-ans*	CBS 578.94	*Infectoriae*	*Triticumaestivum*	Argentina	FJ214834	LR134183	FJ214930	NR_136030	-	-
*A.vaccariicola*	CBS 118714	*Gypsophilae*	*Vaccariahispanica*	USA	KC584147	KC584439	KC584697	KC584224	KC584315	-
*A.vignae*	YZU 171714	*Helianthiinficientes*	*Vignaunguiculata*	China	OK094678	OL763423	OL763421	OL739889	-	-
*A.yamethinen-sis*	YZU 231739	*Alternaria*	*Helianthusannuus*	Myanmar	OR963610	PP179253	OR963614	OR889008	-	-
*A.zantedesch-iae*	CBS 124113	*Ulocladioides*	-	-	KF533900	-	-	-	-	-
*Cicatriceasalina*	CBS 302.84	-	*Cancerpagurus*	North Sea	JN383467	KC584450	KC584709	JN383486	-	JQ671766
*Stemphyliumbotryosum*	CBS 714.68	-	*Medicagosativa*	Canada	OR269991	-	KC584729	NR_163547	NG_069738	-

The phylogenetic analysis was conducted through Maximum Likelihood (ML) and Maximum Parsimony (MP) methods using MEGA11 v.11.0.13 (Tamura et al. 2021). The best-fit evolutionary model was determined automatically by MEGA11 software. The ML analysis was conducted using heuristic searches consisted of 1000 step utilizing the Nearest-Neighbour-Interchange (NNI) algorithm with a Neighbour-Joining starting tree automatically generated. Whereas for the MP analysis, the Tree-Bisection-Regrafting (TBR) algorithm was applied. One thousand (1000) bootstrap replications were conducted to evaluate the generated MP trees robustness. *Cicatriceasalina*CBS 302.84 and *Stemphyliumherbarum*CBS 191.86 were used as outgroup taxa.

## ﻿Results

### ﻿Phylogenetic analyses

**Figure 2.**
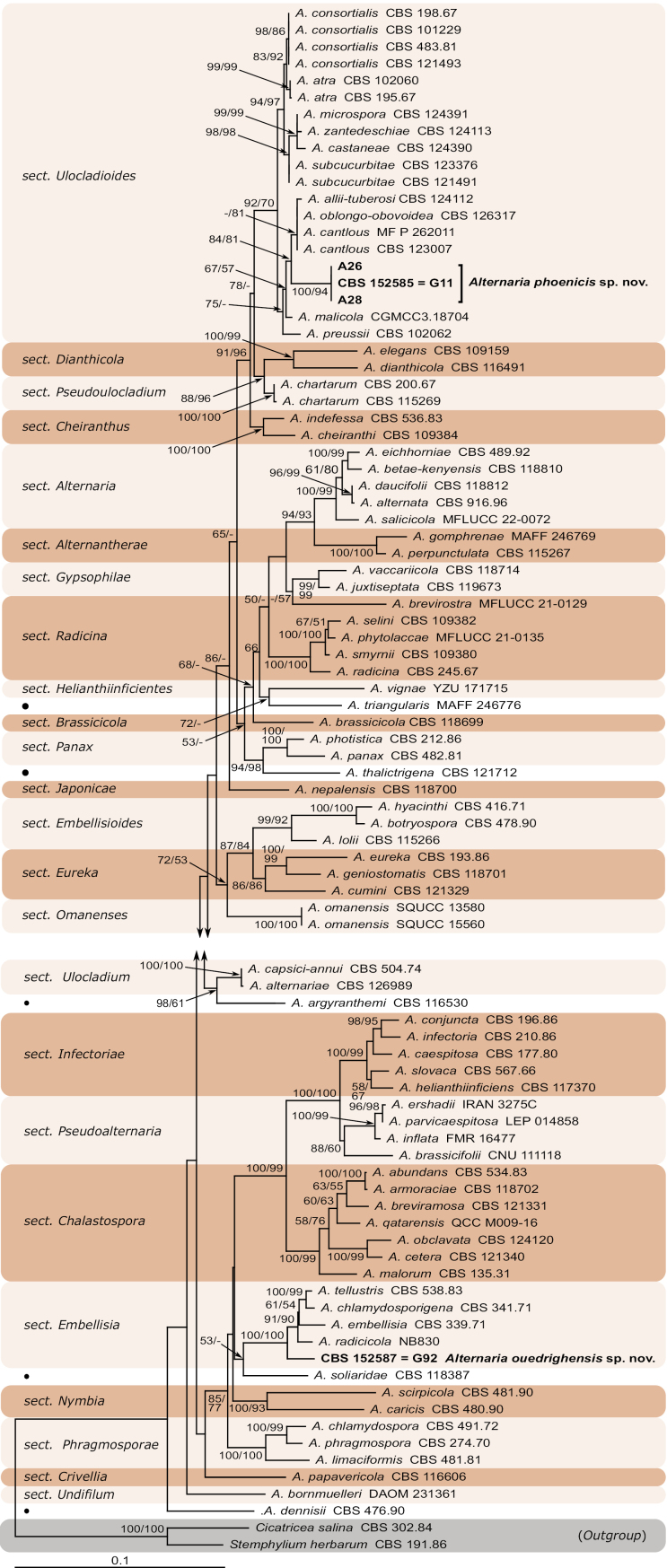
Phylogenetic tree based on the maximum likelihood analysis of *Alternaria* species inferred from combined LSU, ITS, *GAPDH*, *RPB2* and *TEF1*. Maximum likelihood (ML) and maximum parcimony (MP) bootstrap values (≥ 50%) given at the nodes (ML/MP) are computed at from 1000 replicates. The tree is rooted to *Cicatriceasalina* (CBS 302.84) and *Stemphyliumherbarum* (CBS 191.86). The novel species are highlighted in bold. The monotypic lineages are indicated by black dots.

The PCR amplification of the LSU, ITS, *GAPDH*, *RPB2*, *TEF1* and *ATPase* regions yielded DNA fragments of about 1200, 600, 580, 950, 300 and 1200 bp, respectively. Given the lack of the *ATPase* sequences for several species of the *Alternaria* genus and the majority of the species in the *Ulocladioides* sections, this marker has been discarded from the phylogenetic analysis. Those, the concatenated LSU, ITS, *GAPDH*, *RPB2*, and *TEF1* datasets consisted of 90 strains corresponding to 78 species and two outgroup taxa. The alignment contained 2915 characters of which 2031 were constant, 23 were excluded, 161 were variable and parsimony-uninformative and 700 were parsimony-informative. Maximum parsimony (MP) analyses of combined dataset produced a single most parsimonious tree (score = 3577, CI = 0.327, RI = 0.684 and HI = 0.673), which resulted in the identification of the strains. Furthermore, maximum likelihood analyses on concatenated dataset yielded a phylogenetic tree (Fig. 2), which was similar with maximum parsimony tree in terms of either major topology or results. So, it was chosen for the phylogeny demonstration. Alignment and phylogenetic trees were deposited at TreeBASE (ID: 31850).

In the phylogenetic analysis, all the clades corresponding to the *Alternaria* sections were well resolved. Of these, 2 clades corresponding to the sections *Ulocladioides* and *Embellisia* encompassed the strains of this study. The isolates G11, A26 and A28 formed independent well-supported subclade with high bootstrap support (100% ML and 94% MP; Fig. 2) within the section Ulocladioides and were considered to represent a distinct species, which was described here as *Alternariaphoenicis* sp. nov. The strain G92 clustered within the section Embellisia with a high boostrap support (100% ML and 94% MP; Fig. 1), but was phylogenetically different from the closest species within the section. It represented a further distinct species, which was described here as *Alternariaouedrighensis* sp. nov. (Fig. 2).

## ﻿Taxonomy

### ﻿ Alternaria phoenicis

Taxon classificationFungiPleosporalesPleosporaceae

Y. Djellid, A. E. Mahamedi, F. Lamghari & A. Berraf-Tebbal sp. nov.

81F77603-8411-5874-9CC8-D9BF375059FD

856854

Fig. 3

#### Type.

Algeria • Ghardaia Province (32°10'18.174"N, 3°34'56.6976"E), on symptomatic leaflet and rachis of *Phoenixdactylifera* L., 2017, Y Djellid, (MEND-F-1166, holotype), ex-type culture CBS 152585.

**Figure 3.**
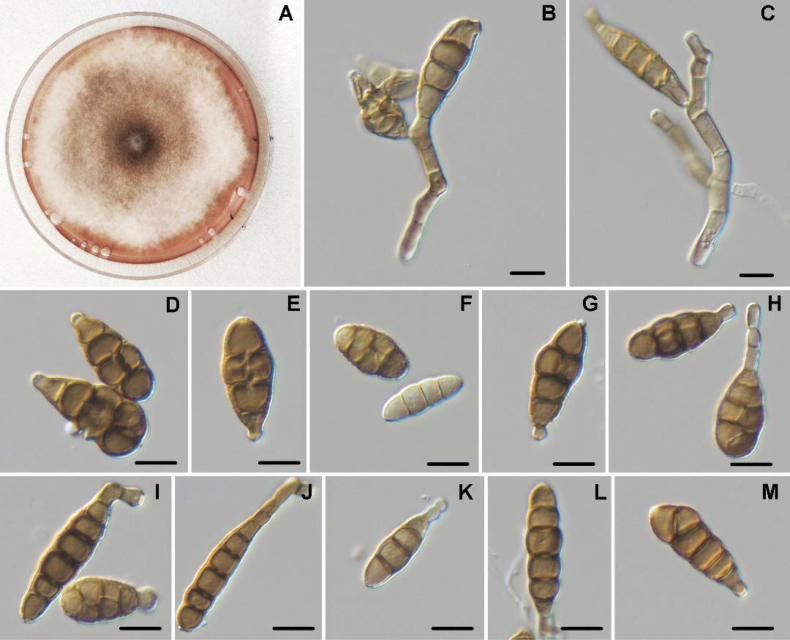
Morphology of *Alternariaphoenicis*. Colony on PDA after 7 days at 25 °C (A); Conidiophores and conidiogenouse cells (B, C); Conidia (D–M). Scale bars: 10 μm.

#### Etymology.

Named after the host genus (*Phoenix*) from which the fungus was isolated.

#### Description.

***Colonies*** on PDA reaching 75 mm diam. after 7 d at 25 °C, circular, cottony with dense hyphae, off-white to light grey in the center, reverse buff to dark brown in the center. Minimum temperature for growth 5 °C, optimum 25 °C, maximum 37 °C. On Potato dextrose agar (PDA; Fig. 3), ***conidiophores*** arising directly from lateral of aerial hyphae, straight or curved, geniculate, smooth-walled, with up to 5–septate, unbranched or with up to two branches, pale brown; ***Conidia*** solitary, subcylindrical to obclavate, (18.1–) 21.4 – 29.1 (–38.8) × (7.4–) 9.7 – 12.8 (–14.8) μm, (av. 25.3 ± 3.9 × 11.2 ± 1.6), non-beaked with a narrow base, light brown, with some darkened middle transverse septa, 3–6 transverse septa, and 0–1 longitudinal or oblique septa per transverse segment; these primary conidia produce secondary conidiophores that consist in a subapical extension from the conidial body. ***Sexual morph*** not observed.

#### Notes.

Phylogenetically, this species grouped within Ulocladioides section but was different from the closest species (*A.malicola*, *A.preussii* and *A.cantlous*) in a distinct lineage with 100% ML / 94% MP statistical support. *Alternariaphoenicis* sp. nov. is different from its sister species *A.malicola*, *A.preussii* and *A.cantlous*, based on sequences derived from five loci (Fig. 2). After conducting a nucleotide pairwise comparison as recommended by Jeewon and Hyde (2016), the present species can be distinguished from the closet species *A.malicola*, *A.preussii* and *A.cantlous*. Based on *GAPDH*, *RPB2* and *TEF1* genes, *A.phoenicis* sp. nov. has 7 bp differences (2%, no gap) in *GAPDH*, 1 bp (1%, no gap) in *RPB2* and 29 bp (7%, 6 gaps) in *TEF1* when compared to *A.malicola*. *Alternariapreussii* presents 5 bp differences (2%, no gap) in *GAPDH* and 11 bp (2%, no gap) in *RPB2*. However, *A.cantlous* shows 1 bp difference (1%, no gap) in *RPB2* and 29 bp (11%, 6 gaps) in *TEF1*. Morphologically, *A.phoenicis* (Fig. 3) can be distinguished by having narrower conidia (7.4–14.8 µm) compared to the three closely related species: *A.cantlous* (7.4–14.8 µm), *A.preussii* (13.0–13.7 µm), and *A.malicola* (8–16 µm). In terms of length, its conidia are shorter than those of *A.cantlous* (24–36 µm) but longer when compared to *A.preussii* (18.3–20.4 µm). However, the conidial length of *A.malicola* (16–35 µm) is comparable to that of *A.phoenicis* (18.1–38.8 µm). Regarding the conidial septation, *A.phoenicis* is characterized by multiple transverse septa (up to 6). In contrast, its closely related species exhibit fewer transverse septa, up to four in *A.canlous* and up to three in both *A.preussii* and *A.malicola*. Additionally, *A.phoenicis* has the fewest longitudinal septa (0–1), compared to *A.preussii* (1–2), *A.malicola* (1–5), and *A.canlous* (0–2) (Runa et al. 2009; Wang et al. 2010; Dang et al. 2018).

### ﻿ Alternaria ouedrighensis

Taxon classificationFungiPleosporalesPleosporaceae

, A. Berraf-Tebbal, A. E. Mahamedi, F. Lamghari, E. Hakalova & Y. Djellid sp. nov.

48EB7EF0-1DD2-585E-B318-2928F954C490

856855

Fig. 4

#### Type.

Algeria • Biskra Province (34°44'16.0152"N, 5°22'10.1064"E), on symptomatic leaf of *Phoenixdactylifera* L. 2017, Y Djellid (MEND-F-1168, holotype), ex-type culture CBS 152587.

#### Etymology.

Named after the valley of Oued Righ from which the fungus was collected.

#### Description.

**Figure 4.**
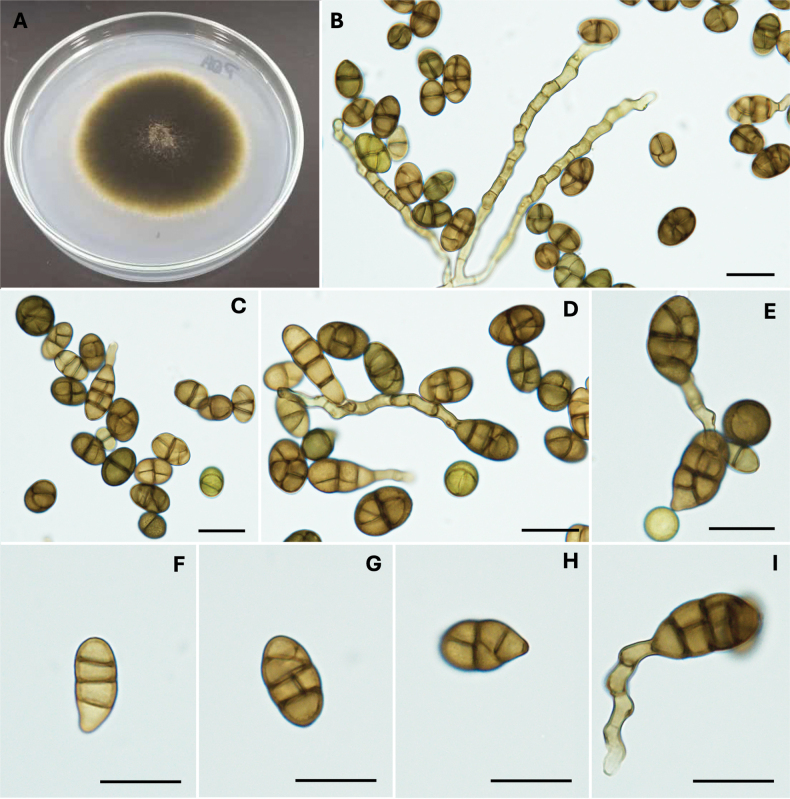
Morphology of *Alternariaouedrighensis*. Colony on PDA after 7 days at 25 °C (A); Conidiophores and conidiogenous cells (B, C); Conidia (D–M) Scale bars: 10 μm.

***Colonies*** on Potato dextrose agar (PDA) reaching 51 mm diam. after 7 d at 25 °C, circular with concentric zonation of the growth, cottony with dense hyphae, dark green, reverse dark brown, with a white halo at the edge. Minimum temperature for growth 5 °C, optimum 25 °C, maximum 37 °C. On PDA media (Fig. 4), ***conidiophores*** arising directly from lateral of aerial hyphae, straight or curved, geniculate sympodial proliferation, verruculose thick-walled, with up to 12–septate, unbranched or with up to three branches, light to dark brown; ***Conidia*** solitary, ovoid to subcylindrical, (11.4–) 15.3 – 17.7 (–24.1) × (7.7–) 9.9 – 10.9 (–12.9) μm (av. 16.5 ± 3.4 × 10.4 ± 1.4), light brown to dark, rigid, and thickened transverse septa, 1–3 transverse septa, and 0–1 longitudinal or oblique septa per transverse segment; these primary conidia produce secondary conidiophores that consist of a subapical extension from the conidial body. ***Sexual morph*** not observed.

#### Note.

Phylogenetically *A.ouedrighensis* formed a sister branch with *A.embellisia*, *A.chlamydosporigena*, *A.radicicola* and *A.tellustris* in Embellisia section with 100% ML/100% MP bootstrap support. *Alternariaouedrighensis* sp. nov. is different from its sister species *A.radicicola*, *A.embellisia* and *A.tellustris* based on sequences derived from five genes (Fig. 2). After conducting a nucleotide pairwise comparison as recommended by Jeewon and Hyde (2016), the present species can be readily distinguished from the closet species *A.radicicola*, *A.embellisia* and *A.tellustris* constructed on any of the LSU, ITS, *GAPDH*, *RPB2* and *TEF1* genes, which has 3 bp difference (1%, no gap) in the ITS region, 6 bp (2%, no gap) in *GAPDH*, 16 pb (2%, no gap) in *RPB2* and 15 bp (11%, 14 gap) in *TEF1* when compared with *A.radicicola*, 1 bp (1%, no gap) in LSU, 6 bp (2%, no gap) in ITS, 24 bp (4%, 1 gap) in *GAPDH*, 17 bp (2%, 1 gap) in *RPB2*, and 17 bp (11%, 13 gaps) in *TEF1* when compared with *A.embellisia*, and 1 bp (1%, no gap) in LSU, 3 bp (1%, no gap) in ITS, 12 bp (2%, 1 gap) in *GAPDH*, 17 bp (2%, no gap) in *RPB2* and 13 bp (9%, 14 gaps) in *TEF1* with sister species *A.tellustris*.

Morphologically, *A.ouedrighensis* (Fig. 4) is distinct from the closest species *A.embellisia* in conidial body size. *Alternariaouedrighensis* has conidia shorter and wider (11.4–24.1 × 7.7–12.9 μm; av. 16.5 ± 3.4 × 10.4 ± 1.4 µm) than those of *A.radicicola* (20–38 × 7–10 µm; Bessadat et al. 2025) and *A.embellisia* (19.18–36.2 × 2.55–5.74 µm; av. 12.64 × 4.34 µm; Delgado Ortiz et al. 2019). In addition, the conidia of *A.ouedrighensis* present fewer transverse septa (1–3 transverse septa) than those of *A.radicicola* (3–5 transverse septa) and *A.embellisia* (2 – 6 transverse septa). However, *A.ouedrighensis* presents fewer longitudinal septa (0–1 septum) compared to *A.embellisia* (1 – 2 septa).

## ﻿Discussion

In this study, two new species of *Alternaria*, *A.phoenicis* and *A.ouedrighensis*, have been identified within the sections *Ulocladioides* and *Embellisia*, respectively. These species were characterized and illustrated through comprehensive morphological studies and a detailed polylocus phylogenetic analysis, which provides robust support for their classification within the genus. Both species are associated with black spot and blight diseases symptoms on date palm (*Phoenixdactylifera* L.). These diseases present a range of symptoms that can significantly compromise the health and productivity of this host tree. Black spot disease typically manifests as dark, circular lesions on the leaves, often surrounded by a yellow halo, which may merge to form larger necrotic areas. This condition can lead to premature fall of the leaves, thereby substantially reducing the photosynthetic capacity of the plant (Elmer and Pscheidt 2014). While the blight disease symptoms are characterized by rapid wilting and dieback of fronds. The affected leaves exhibit browning that typically initiates at the tips and progresses inward, leading to significant tissue necrosis and overall leaf decline, which can result in wilting and dieback. These conditions can impact the structural integrity and physiological function of the date palm (Namsi et al. 2019).

*Alternariaphoenicis*, the newly described species, forms a clearly separate cluster within the section Ulocladioides, in the multi-locus phylogenetic trees derived by analyses of a concatenated DNA sequence dataset. This section encompasses a diverse group of species recognized for their significant ecological roles and potential agricultural impacts. They are mostly known as saprotrophs on a variety of host substrates as well as opportunistic human pathogens (Runa et al. 2009; Lawrence et al. 2016; Gannibal and Gomzhina 2024). The Ulocladioides section was introduced in 2013 by Woudenberg et al. to accommodate species previously classified under Ulocladium section. Thus, the Ulocladioides section included 20 species typified by *Alternariacucurbitae*. Recently, Gannibal and Gomzhina (2024) assessed the species boundaries within the Ulocladioidessectionby using multilocus phylogenetic analysis based on the genealogical concordance phylogenetic species recognition (GCPSR) principle. They also utilized the coalescent-based model Poisson tree processes (PTP, mPTP) and evaluated for the presence of recombination. As a result, they suggested to eradicate nine species by joining four other species. *Alternariaatra* and *A.multiformis* were united into the single species *A.atra*. Five species, *A.brassicae-pekinensis*, *A.consortialis*, *A.cucurbitae*, *A.obovoidea*, and *A.terricola*, were combined in the species *A.consortialis*. *Alternariaheterospora* and *A.subcucurbitae* were combined into one species, *A.subcucurbitae*. *Alternariaaspera*, *A.chartarum*, *A.concatenata*, and *A.septospora* were combined into a single species, *A.chartarum*. Morphologically, species within this section can be identified by their short, geniculate conidiophores, with sympodial proliferations and obovoid, non-beaked conidia, with a narrow base, single or in chains (Woudenberg et al. 2013; Li et al. 2023).

The second new species *A.ouedrighensis* is introduced and classified in section Embellisia within the genus *Alternaria*. This section was established to include previously described species under the genus *Embellisia* (Lawrence et al. 2012). It is currently limited to only four species: *A.embellisia* Woudenb. & Crous, the type species, along with *A.chlamydosporigena* Woudenb. & Crous, *A.tellustris* (E.G. Simmons) Woudenb. & Crous and *A.radicicola* Bessadat & Simoneau (Woudenberg et al. 2013; Li et al. 2023; Bessadat et al. 2025). Phylogenetic analyses revealed the close relationships among these four species and highlight their evolutionary ties to other sections of the *Alternaria* genus. Notably, these species exhibit consistent morphological traits, including thick, dark, and rigid conidial septa, along with a limited presence of longitudinal septa, which serve as identification keys. Additionally, members of this section have been recognized as pathogens that impact various vegetable crops, particularly tomato and garlic (Simmons 2001; Woudenberg et al. 2013). Although *A.ouedrighensis* is currently represented by a single isolate, its recognition as a new taxon remains valid, consistent with previous studies (Crous et al. 2015; Lücking et al. 2021), that have formally described novel species based on distinct phylogenetic placement and unique morphological characteristics. Consequently, it is necessary to set up larger surveys and isolations that include more phoenicical production areas to better understand the diversity and intraspecific variability within *Alternaria* species.

The identification of these new species not only enriches our understanding of the diversity within the *Alternaria* genus but also emphasizes the necessity for effective management strategies to minimize the impact of this genus on plant health and productivity.

## Supplementary Material

XML Treatment for Alternaria phoenicis

XML Treatment for Alternaria ouedrighensis

## ﻿Additional information

### Use of AI

No use of AI was reported.

### Author ORCIDs

Youssef Djellid https://orcid.org/0009-0007-6833-5439

Alla Eddine Mahamedi https://orcid.org/0000-0002-9744-8973

Milan Spetik https://orcid.org/0000-0001-7659-8852

Eliska Hakalová https://orcid.org/0000-0002-5433-8993

Ales Eichmeier https://orcid.org/0000-0001-7358-3903

Micael Ferreira Mota Gonçalves https://orcid.org/0000-0003-2295-3374

Fouad Lamghari https://orcid.org/0009-0002-2789-2240

Maryam Ali Saeed Mohamed Al Hmoudi https://orcid.org/0009-0005-9207-4924

Akila Berraf-Tebbal https://orcid.org/0000-0001-8517-8542

### Data availability

All of the data that support the findings of this study are available in the main text.
